# Prof. Hamilton’s Introductory Lecture

**Published:** 1850-02

**Authors:** 


*Prof. Hamilton's Introductory Lecture.*—A part of the Lecture by Prof. Hamilton, introductory to his course of lectures now in progress, was devoted to a sketch of the life of N. T. Otis, Jr., a pupil of Prof. H., who fell a victim to epidemic dysentery during the last summer. This portion of the lecture has been published, by request, in a pamphlet form. As a tribute to the memory and merits of the deceased, it reflects honor on the author, and is, moreover, replete with sentiments which should serve as incentives to high aspirations and unremitting industry.

